# Comparison of data science workflows for root cause analysis of bioprocesses

**DOI:** 10.1007/s00449-018-2029-6

**Published:** 2018-10-31

**Authors:** Daniel Borchert, Diego A. Suarez-Zuluaga, Patrick Sagmeister, Yvonne E. Thomassen, Christoph Herwig

**Affiliations:** 1 Exputec GmbH, Mariahilferstraße 147/2/2D, 1150 Vienna, Austria; 2 Intravacc, Antonie van Leeuwenhoeklaan 9, 3721 MA Bilthoven, The Netherlands; 3 Research Area Biochemical Engineering, Vienna University of Technology, Gumpendorferstrasse 1a, 1060 Vienna, Austria

**Keywords:** Root cause analysis, Data science, Data analysis, Feature based analysis, Raw data analysis

## Abstract

Root cause analysis (RCA) is one of the most prominent tools used to comprehensively evaluate a biopharmaceutical production process. Despite of its widespread use in industry, the Food and Drug Administration has observed a lot of unsuitable approaches for RCAs within the last years. The reasons for those unsuitable approaches are the use of incorrect variables during the analysis and the lack in process understanding, which impede correct model interpretation. Two major approaches to perform RCAs are currently dominating the chemical and pharmaceutical industry: raw data analysis and feature-based approach. Both techniques are shown to be able to identify the significant variables causing the variance of the response. Although they are different in data unfolding, the same tools as principal component analysis and partial least square regression are used in both concepts. Within this article we demonstrate the strength and weaknesses of both approaches. We proved that a fusion of both results in a comprehensive and effective workflow, which not only increases better process understanding. We demonstrate this workflow along with an example. Hence, the presented workflow allows to save analysis time and to reduce the effort of data mining by easy detection of the most important variables within the given dataset. Subsequently, the final obtained process knowledge can be translated into new hypotheses, which can be tested experimentally and thereby lead to effectively improving process robustness.

## Introduction

The holistic assessment of data from integrated biopharmaceutical production processes became more and more popular within the last decade [1]. Deviations within process parameters and raw material attributes must be collected in a first step as these have a high impact on manufacturing costs. Additionally, their potential effects on drug product need to be evaluated even after a few experimental runs [2, 3]. Furthermore, it is preferred to identify those events from which we are able to learn most. The prominent tool for performing such an analysis is root cause analysis (RCA). RCA is a technique used to detect the origin of deviations in response parameters within a dataset [4].

RCA is widely used in the pharmaceutical industry to identify the influence of critical process parameters (CPPs) [5–7] on critical quality attributes (CQAs). Furthermore, key performance indicators (KPIs) during process scale up [8, 9] and process analytical technologies (PAT) [10, 11] are currently evaluated with RCA. The variable of interest, for which the source of variance should be identified, is called the dependent variable or response variable. The overall aim of the RCA is to identify the root cause of deviations in the response variables using all the existing process information, ideally all recorded variables. Nevertheless, a high number of inadequate RCA have been observed by the FDA within the last years. The reasons for that are summarized in a current FDA letter [4]. The most important statement from this article is that it is very important to choose only those events where it is believed that there is a significant amount of learning to be gained. It can be seen that it is essential to find the best model that can explain most of the response variance using few independent variables.

To perform an adequate RCA, two major tools are currently used in industry.

- Raw data analysis (RDA) [8]: The raw data are of main importance during the entire analysis. This technique is designed to use the recorded data holistically.
- Feature-based approach (FBA) [12, 13]: The raw data are used to detect deviations within a time series process. These deviations are extracted from the recorded data and used as single observations for further analysis.

Both tools can be used to identify the (C)PPs responsible for deviations of a certain CQA. To select the best approach for the given dataset, the content of the dataset is of major importance. Currently, it is very difficult to select the best approach to analyze the actual dataset integrally. At present, there are no comparisons of both approaches holistically and comprehensively making it difficult to select the best approach for a given dataset.

Statistical and process knowledge is required to perform a RCA [14]. Although process knowledge and basic statistical know-how is available, a fundamental statistical training is almost missing in biopharmaceutical companies. Therefore, external companies, with exactly this knowledge, are usually commissioned to perform the analysis. One of the biggest issues in doing this is that the external company is often lacking the required process knowledge and requires help from a process expert to perform a comprehensive RCA.

Here, we present a comprehensive roadmap for performing a RCA that will not only allow to reduce time, but can also be followed by personnel with limited statistical and/or process knowledge. Additionally, we demonstrate the differences and similarities between RDA and FBA, as well as their advantages and disadvantages. The evaluation was conducted using the same dataset for both techniques and used the root mean square error of cross validation (RMSECV) of the performed partial least square (PLS) regression model to identify the best performing model. This case study was recently conducted at a leading R&D facility for vaccine development.

Furthermore, the demonstrated RCA workflow can be applied to any kind of biopharmaceutical batch or fed-batch process. To evaluate such processes, time series data as well as one-point measurements should be tracked and included into the analysis. This new methodology guides the analyst through the different process steps and result evaluation, independently. The final gained process knowledge should then be used to extract reasonable events to do a comprehensive data analysis with simple and state-of-the-art statistical tools.

## Materials and methods

### Data

The dataset used to evaluate RDA and FBA was derived from experiments on Vero cell culture followed by poliovirus production. The dataset consisted of 40 bioreactor operations used to produce poliovirus type 2 (Sabin) within an animal component-free media environment. Vero cells growing adherent to microcarriers were used as host cell line to produce the poliovirus. The fermentation process was split into two process phases, a cell culture and virus production phases. The response variable selected for the investigation (D-antigen concentration) is measured once at the end of the process and is considered as key performance indicator of the upstream process (USP) [15].

### Software

Two commercially available software tools were used to perform the RCA. SIMCA version 13.0.3.0 (Umetrics AB, Umea, Sweden) was used for RDA. To perform FBA, inCyght^®^ Web version 2018.04 (Exputec GmbH, Vienna, Austria) was used. The required uni- and multivariate statistical analysis tools were already implemented in these software tools. Prior to data analysis with SIMCA the data were preprocessed with inCyght^®^, MS Excel 2016 (Microsoft, Redmond, WA) and Python 3.3 (Python Software foundation, https://www.python.org/).

### Statistical methods

PCA and PLS were performed as standard tools. A sevenfold cross-validation was applied for the selection of the best subset of the PLS. The subset with the lowest RMSCV is regarded as the best subset. The lower the RMSCV, the better is the prediction and the better the model [16]. Cross-validation is a procedure, at which the dataset is split in $n$ equal parts. The idea behind this is that an amount of $n-1$ of these parts are used to predict the remaining one, where resulting residual error is the RMSCV. Both methods are standard tools within the used software tools.

## Results

**Fig. 1:**
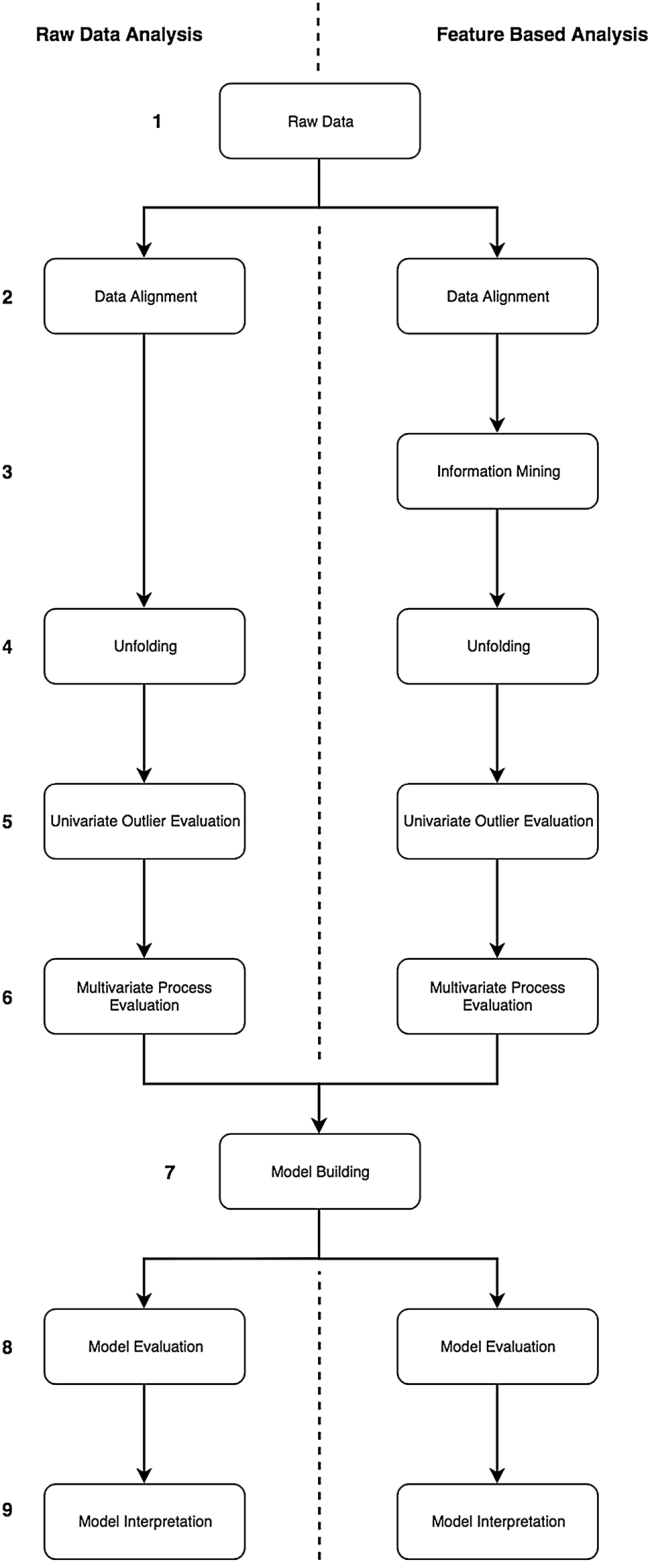
Root cause analysis (RCA) workflows. The left side represents the RDA approach (A) and the right side represents the FBA approach (B). Each step is displayed as an individual square. If both approaches use the same methodology, then one square is displayed for both applications. However, if there are differences in the analysis or in the generated plots a separate square for each approach is displayed. In general, it can be seen, that for both applications the same steps are required, except the step 3 “Information Mining” which is a unique step within the FBA

Prior to starting a RCA, the response must be identified and the analysis concept has to be chosen. In the hereafter section the common approaches for RCA, RDA, and FBA, will be evaluated. A comparison of the RCA workflows of the RDA and the FBA can be found in Fig. 1, on the left and right side, respectively. The specific steps in these workflows are described independently in this section. A comprehensive evaluation of these steps and a joint application is shown in “Discussion”.

### Raw data

To perform data analysis, the batches and the corresponding data need to be selected and collected. This data accumulation step is equal for both approaches (Fig. 1). The step represents the data generation and mining out of different devices which are used during the biopharmaceutical process. Such a process typically has different sources of data which are finally collected in a holistic dataset. Basically it can be distinguished between, one point and time series data. To evaluate the properties of each data source, consider Table 1.

**Table 1:** Summary of different data sources, occurring within a standard biopharmaceutical production process

Characteristic	Data source	Property	Example/s
Initial recorded data	One point measurement	Time independent (*t* = 0)	Starting volume Inoculum density
Online data	Time series	High-frequent recorded data generated during process	Data from sensor and PAT tools, typically connected to SCADA system
Offline data	Time series	Low-frequent recorded data generated during process (one measurement/day)	Bio-profile measurements such as sugars and metabolites analysis
Harvest data	One-point measurement	Almost time-independent (*t* = end of process)	Product titer Impurity concentration

Two different types of data sources are typically distinguished, one point and time series data. The certain sources mainly differentiate in the property of time-dependent or -independent

The finally obtained dataset consists of one-point measurements that may be time independent and frequently recorded time dependent data.

### Data alignment

The second step in the workflow is the data alignment. In the obtained dataset the data is not yet structured and evaluating the data is impossible. The data needs to be structured. First, the exact start and end times need to be evaluated and certain process phases have to be defined. Next, the data can be sorted and listed to allow plots to be generated. Alternatively, the structured data can be imported in software tools for data analysis.

Initial data structuring was similar for both the RDA and the FBA approach. In the studied bioprocess two phases can be distinguished. The first phase is cell culture. In this phase cells are expanded to the desired amount of cells which will serve as production host for virus propagation. The second phase is the virus production phase. The end of the virus production phase is equal to the end of the total process. For both phases, the start and end time points were aligned. In other words, time series data were split into a cell culture phase and a virus production phase. Once alignment is performed, the initial dataset is ready for analysis and can be imported to the analysis software of choice (Fig. 1). The current data set was subsequently uploaded into inCyght^®^ database. Starting at this point, the presented workflow splits up. This illustrates that while the benefit of this step is the same for both approaches, there are differences within the way data is imported and plotted.

In the case of RDA the holistic dataset was exported to Excel from the inCyght^®^ database. The resulted table is already structured in the case of one point and time series data. The final process phase allocation was done with a house intern Python script. Finally, the data table was imported into SIMCA.

In the case of FBA, the data are selected from the inCyght^®^ database and different kind of plots for the time series data and one point measurements can be made. The generated time series overlay plots can be aligned concerning the process phases. This plot can be further used to identify deviations for certain process variables.

### Information mining

Information mining is only done in case of FBA and needs to be done prior to unfolding. For FBA this means that certain time series data is described by new variables such as rates and yields. Each biopharmaceutical production process is unique and a lot of error sources are often overseen before starting this analysis. Overlay plots of each time series variable should be made to detect possible deviations, consider “Data alignment”. Additionally, to draw comprehensive information out of certain time series variables, like cell or metabolite concentrations, specific rates and yields are calculated. In this way, the detected deviations within the time series can be described by single values (or features). If for instance the nitrogen supply for a virus culture process always starts at a different time point, the mined information of the nitrogen variable is the process time at which the nitrogen supply starts. The newly generated variables can be evaluated and added to the dataset, consider “Unfolding”. This work package was solely performed within the FBA workflow, because RDA uses only the raw data information.

### Unfolding

**Fig. 2:**
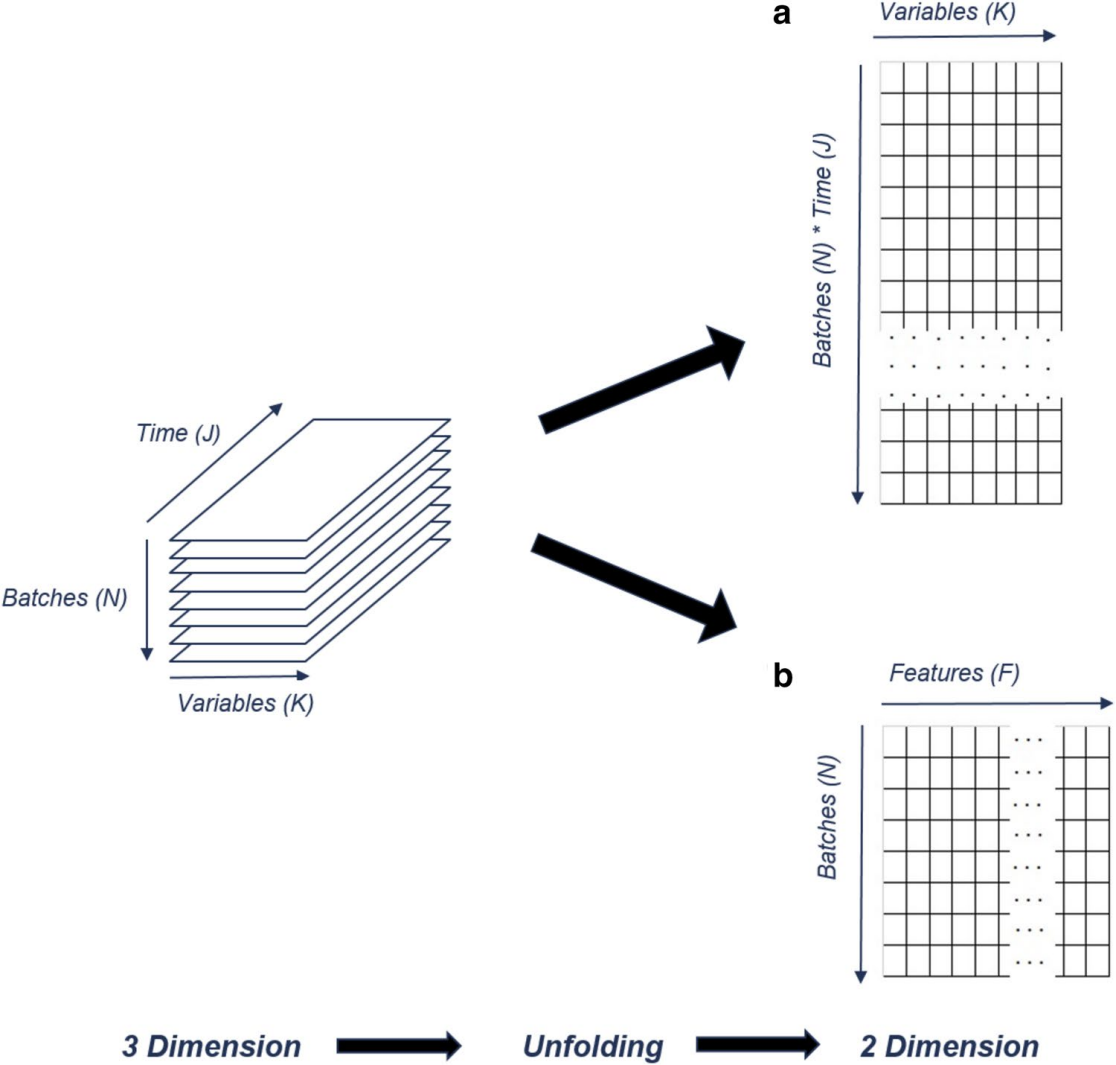
Unfolding procedure to reduce the three dimension matrix to a two dimensional matrix. The initial dataset has batches (*N*) on the *y*-axis, variables (*K*) on the *x*-axis and time (*J*) on the *z*-axis in a three dimensional space. The aim of the unfolding is the same for both approaches, but the resulting two dimensional dataset is different. The final data set which will be used for the RDA (**a**) has a shape of NxJ rows with *K* columns. Each raw contains data points xijk from a single batch observation. The terminal data set which will be used for the FBA (**b**) has a shape of *N* rows with features (*F*) columns. A feature is a certain data information within a time series data. The number of features depends on the initial and final one point measurements as well as strongly on the amount of extraction done by the data scientist, which are evaluated by detecting deviations within a time series overlay plot

Almost each multivariate data analysis tool requires a certain data format. To generate this format, the current available three-dimensional dataset with a typical shape of batches (*N*) × variables (*K*) × time (*J*) has to be reduced to a two-dimensional dataset. This dimension reduction step is called dataset unfolding. Although the same initial three-dimensional data matrix shape is used for the RDA and the FBA, the unfolding step is quite different. Figure 2a, b displays the unfolding procedure and addresses the differences in the resulting matrices.

It can be seen that after unfolding the resulting data table looks different for RDA and FBA (Fig. 2). Figure 2a displays the unfolding procedure performed for the RDA approach. The two-dimensional data table, which is further used for uni- and multivariate data analysis (MVDA), has *N* × *J* rows and *K* columns. *N* represents the number of batches, *J* the number of time points and *K* the number of time series variables. It can be seen that more observations (rows) than variables (columns) are present. Within this kind of table, all recorded data are used holistically since each row contains data points ${x}_{ijk}$ from a single batch timepoint observation.

The shape of the unfolded data table for FBA, shown in Fig. 2b, indicates that there are usually less observations (rows) than variables (columns). Although the case of less observation than variables is not very common in statistical analysis. We assume that five observations are enough to represent the current samples’ population holistically. Therefore, it is possible to use the current data set for this kind of analysis. The number of rows is equal the number of the batches. The variables list, containing the one-point measurements, is supplemented with the feature variables (*F*) that have been extracted from the time series during the information mining step. If deviations within certain time series overlay plot were observed, these events were extracted. These extracted values were used to substitute the time series variable comprehensibly to reduce the variable time for the FBA. The resulting data table includes initial available one-point measurements, compare Table 1 and the newly extracted features. This extended dataset will reflect all the potential sources of variance within the current process.

### Univariate data set evaluation

So far the data were inspected quantitatively and no qualitative investigations took place. To perform a comprehensive and representative data analysis, the data integrity must be evaluated. The data has to be checked for completeness to ensure that for each batch a valid variable is available. The aim of this step is to provide a complete gapless data matrix, which is mandatory for multivariate data analysis. Variables with missing data either need to be discarded or when there is only a limited amount of missing data a certain imputation strategy must be followed to fill these gaps. A missing value of a certain variable was imputed by the mean of all the available values from that variable. This strategy is further mentioned as mean imputation. Furthermore, the data has to be checked for outliers, which can be certain observations of a single variable, which do not fit the major population. Due to the fact that the data matrix after unfolding is different in its shape, both approaches use different methods to detect these outliers.

With respect to RDA, each column of the data table is averaged and the standard deviation (STD) is calculated. An overlay plot of a certain variable is generated over time and additionally the calculated average and the upper and lower control limits (CL), which are the $\text{average} +3\text{STD}$and $\text{average}- 3\text{STD}$, respectively, are added to the overlay plot, to facilitate interpretability. If a variable of a certain batch is located outside the CL, it has to be decided whether to exclude the batch or not for further analysis. It must be noted that each variable and batch has to be evaluated independently for validity. The variable average and the CL have to be recalculated each time once a variable is excluded until all remaining variables outside the CL have been evaluated.

**Fig. 3:**
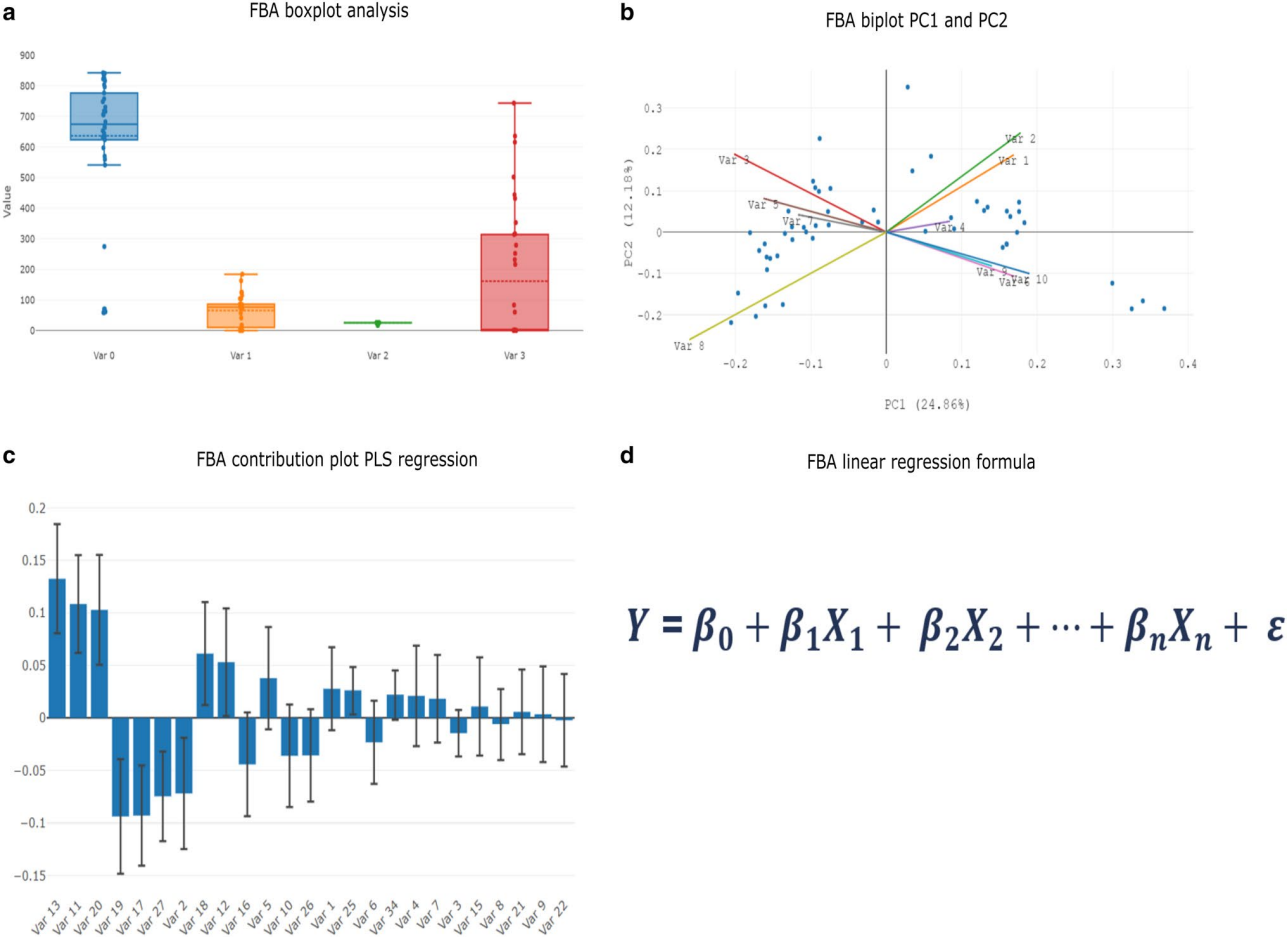
Plot summary of the FBA. **a** Boxplot distribution, of a set of variables, which is used for univariate outlier detection. **b** Biplot of the principal component 1 and 2 of the performed principal component analysis (PCA). This plot gives a hint of the variable relationships within the multivariate space. **c** Result of the partial least square (PLS) analysis. This plot indicates the relation of certain variables onto a response, the direction of the blue bar indicates the direction of the dependency. If the black error bar does not include 0 the variable can be seen as statistically significant influencing the response variable. **d** This formula indicates the final result of the RCA mathematically. *Y* is the response variable, which can be described by the intercept (*β*_0_) and the significant variables (*β*_*n*_*X*_*n*_), where *β*_*n*_ is the slope and *X*_*n*_ the values the certain variable *n* of one of the significant variables out of PLS regression. *ε* represents the variance of the residual error of all other factors not accounted for. We assume that this error is normal distributed with 0 variance and therefore neglected from the analysis

For FBA, a box plot analysis is used to evaluate outliers. Every variable must be investigated and each observed outlier has to be evaluated for validity and meaningfulness. An observation outside the box, aka below the first quartile and above the third quartile of current samples is considered an outlier. Figure 3a represents the boxplot analysis of a set of variables. Furthermore, the box indicates the variable variance and the horizontal line located in the box represents the sample mean. Finally, it is very important to define, if outliers are excluded, accepted or corrected for further data analysis to avoid enforcing a leveraging effect and a misinterpretation of the resulting model.

### Multivariate data set evaluation

Up to this point, the data were evaluated in a univariate manner, which means that every variable was inspected independently. Now, the focus will center on batch-wise evaluation and identification of potential correlations between the variables.

**Fig. 4:**
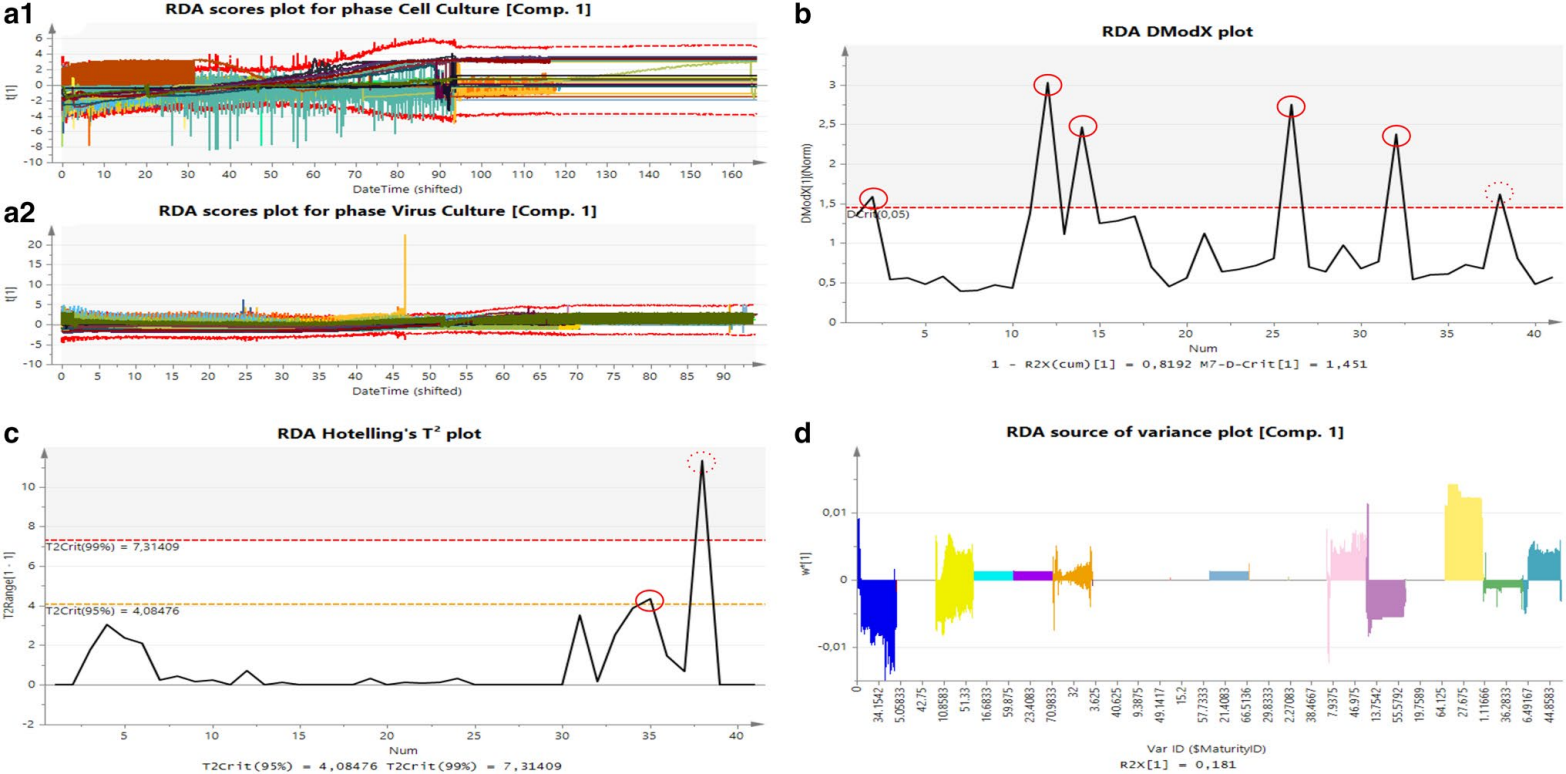
Plot summary of the RDA approach. **a.1** Scores plot of the component 1 of cell and **a.2** virus culture phase. The green dashed line represents the average of all batches. The red line represents the 99% (± 3 Sigma) confidence interval (CI) calculated using all the selected batches. Abnormal batches which are located outside the red line could be identified just within cell culture phase. **b** DModX chart of the batch-level monitoring. This kind of plot is used to identify outlying batches in orthogonal direction, which are located above the model population. If a batch exceeds the red dashed line, representing the 95% CI of the model population, the batch can be stated as different and is marked with a red circle. **c** Hotelling *T*^2^ plot. This plot is used to identify outliers in scores direction, which are batches located next to the model population. Batches larger than the yellow dashed line (95% CI of the model population) can be stated as dubious, while batches located above the red dashed line (99% CI of the model population) can be seen as serious outliers, always marked with a red circle. The most crucial observation is outside the CI in scores and orthogonal direction, red dashed circled run. **d** Source of variance plot. This plot displays the share in total variance of all independent variables used for model building onto the response variable. A high bar graph indicates high influence on the response at this time point. (Color figure online)

Figure 4a, displays the scores plot of a PCA from the RDA approach. This plot displays the selected score value over time for all batches. Scores are the projection of the hyper plane in *x*-direction, which facilitates the identification of the variance within the three dimensional model. A normal batch always stays within the red lines (± 3 sigma from the mean) over the entire evolution, while a batch located outside the red lines indicates a different behavior somewhere in the process. Abnormal batches could only be observed within the process phase cell culture (Fig. 4a.1), while no abnormalities were identified for the process phase virus production (Fig. 4a.2). This result addresses that most fluctuations in batches are present within the cell culture phase and that this phase will potentially influence the response variable significantly. The batch-wise investigation indicates differences of certain batches and facilitates understanding of the process in a holistic manner.

To follow the FBA workflow and to gain a multivariate process understanding of the current data set, a PCA was performed. The PCA enables a separation of signal to noise within the data, by additional dimension reduction. The resulting biplot, Fig. 3b, of the first and second principal components of the PCA, is used to compare the interaction of the different variables to each other. Interactions of variables can be explored in a low-dimensional representation and variables pointing in the same direction are positively correlated. Each set of collinear variables (variables located next to each other within the biplot) has to be evaluated and reduced to just the most meaningful variable. This procedure reduces the current dataset and prevents the problem of collinearity within the next steps, which will lead to misinterpretation of the multivariate model evaluation.

### Regression model building

After uni- and multivariate data set evaluation, the multivariate regression model can be calculated. This regression model displays the relationship between independent variables and a response variable in the multivariate space. In this case a PLS regression was performed using both unfolded datasets and the same response variable was selected.

To identify the best number of latent variables used for PLS calculation a sevenfold cross validation procedure was performed. The RMSCV of each data subset was calculated and the subset with the lowest RMSCV value represented the best model for the present dataset and analysis concept. Table 2 summarizes the PLS model results of both the RDA and FBA approach. It can be seen that the RMSCV of the FBA approach is lower than for the RDA approach. Which means that the obtained subset from the FBA has more power to predict the response variable. Also, potentially more noise is present in the unfolded RDA dataset. Additionally, we observed a difference in the number of variables and the number of significant variables used to build the regression model, while the imputation strategy was the same for both approaches.

**Table 2:** Partial least square regression summary table

	RDA	FBA
RMSCV	26.87	0.84
Number of independent variables used for model building	23	33
Significant variables	11	10
Missing values imputation	Mean imputation	Mean imputation

The RDA uses 23 independent variables to build the model but only 11 of them were significant. The FBA uses 33 independent variables of which 10 were significant.

### Regression model evaluation

In the calculated multivariate regression models many different plots are usually generated. This section will focus on the most important plot types, generated by each system, which are required to identify the root cause on the selected response variable.

The results of the PLS regression using RDA can be seen in Fig. 4b, c. Part b displays the DModX plot. This plot is used to identify outlying batches in orthogonal direction, which are batches distinctly above the model population in a three-dimensional space. The red line (DCrit 0.05) indicates the 95% CI of the model, six out of 36 batches are outside this boundary. The Hotelling’s *T*^2^ plot (Fig. 4c) shows a yellow and red line indicating the 95% and 99% confidence interval, respectively. Batches above the yellow line can be stated as suspect while batches outside the 99% CI can be stated as serious outliers. These batches are located next to the model population. Within this dataset one suspect and one serious outlying batch could be identified. The most critical observation within the entire dataset is a certain batch which is an outlier in orthogonal and scores direction. In our case batch 39 shows that behavior, compare Fig. 4b, c, red dashed circled run. This batch is definitely outside the population and the reason for that needs to be independently evaluated.

The major plot of the performed PLS regression following the FBA approach can be seen in Fig. 3c. The plot displays the PLS model coefficient plot for the selected response. The blue bars indicate the relative impact of a certain variable on the response variable. The error bar indicates the standard error of a certain variable, if the error bar encloses zero, the variable can be seen as non-significant contribution to the model. The coefficients are sorted from the largest one, which is most impacting variable, to the smallest one. Finally, 10 out of 26, have a significant impact onto the response variable.

### Model interpretation

To draw the correct conclusions from the generated plots, each plot has to be evaluated independently. Also all collected information needs to be regarded to interpret the final result.

The source of variance plot displayed in Fig. 4d, depicts the variance caused by each variable. These variables, displayed as with unique colors, have to be evaluated individually. The contributions of the 11 significant variables, identified using PLS, are illustrated in a time-dependent manner providing a good visual overview. However, the interpretation of certain hypotheses, which are defined at the beginning of data collection, will be difficult at this part of the analysis.

The final interpretation of a RCA while following the FBA, is displayed in Fig. 3d. The displayed formula represents the model result. This mathematical formula indicates the dependency of the *Y* (response) onto the model parameters ${\beta _{0~}}\;{\text{and}}\;~{\beta _n}$. ${\beta _{0~}}$is the intercept and ${\beta _{n~}}~$ is the slope of the variable n. The number of n is equal the number of significant variables. To evaluate the PLS coefficient plot, the corresponding values are further abbreviated with ${X}_{n }$. The term of error variance is indicated with $\epsilon$, this term accounts the error of *Y* of all other factors which are not accounted for. It is assumed that the errors are normal distributed around zero with results in 0 sample variance. This is the reason why this term can be neglected from the resulting model. It can be seen, that the generated results can be used to test certain hypotheses. Process knowledge is still required to interpret the results with respect to RCA.

## Discussion

The aim of this case study was to reduce the effort for the commissioned data scientist or the process engineer, while performing a RCA to evaluate the most important variables onto a certain response. Already, Charaniya et al. [14] and the FDA [4], recommended the need for process knowledge when performing multivariate data analysis. Process knowledge will help to evaluate the generated model appropriately, to evaluate the power of the model and to reduce time by selecting the most reasonable variables of the (biopharmaceutical) production process. Beyond the process knowledge, statistical know-how is an essential part for a successful RCA. Indeed, many statistical applications are available to identify the potential influential parameter on a certain response variable in a multivariate space.

### Comparison of RDA and FBA

In this study, we focused on the most prominent applications, PCA and PLS regression. This is because these are common statistical tools in chemical and pharmaceutical industries. Furthermore, the aim of the analysis was to provide a roadmap for RCA and to enable performance of an adequate analysis using well-established techniques. In this article we investigated the RCA using two different approaches, with the aim to address the most important variables qualitatively. The workflow comparison as well as common steps are displayed in Fig. 1. The amount of steps are almost identical yet nearly each step is different in the performed methods. In Table 3 a summary of the advantages and disadvantages of each of these steps is given, further the key purpose of each step is indicated.

**Table 3:** Workflow overview and comparison of advantages and disadvantages of each step

Step	Advantages		Disadvantages		Purpose
	RDA	FBA	RDA	FBA	
1 Raw data	+ Equal start dataset + Comprehensive dataset + Real data information accessible		− Different data sources (time-dependent and -independent) − Data preprocessing − Time effort		Holistic dataset
2 Data alignment	+ Organized dataset	+ Phase alignment	− Difficult plotting opportunity	− More effort	Dataset ready for analysis
3 Information mining	−	+ Unique process information	−	− Process knowledge needed	Further information generation
4 Unfolding	+ All information available	+ Time-independent variables	− Huge data matrix	− Data subset	Dimension reduction
5 Univariate outlier evaluation	+ Fast	+ Detail data overview	− Time-dependent	− Time consuming	Valid data matrix
6 Multivariate process evaluation	+ Holistic process evaluation	+ Collinearity evaluation	− Noisy data handling	− Specific for extracted variables Process knowledge needed for interpretation	Multivariate overview
7 Model building	+ Best variable subset identification + Multivariate dependencies evaluation		− Calculation intensive − Data imputation required		Multivariate regression model
8 Model evaluation	+ Batch-wise evaluation	+ Simple interpretation	− Difficult interpretation	− Variable wise	Overall model interpretation
9 Model interpretation	+ Comprehensive overview	+ Hypotheses testing	− Hypotheses testing	− Statistical know-how required	Detail model interpretation

The purpose of each step is summarized in the last column and shows the motivation behind each step. Each row represents the step in the workflow shown in Fig. 1, and the first columns pointed to the advantages of each approach, the second highlights the disadvantages of both approaches and last columns summarizes the purpose of each step

Nearly every step of the two investigated approaches is rather different, although the purpose of each step is always the same. Both approaches result in the detection of root cause to explain the variance of a response variable.

In summary, for the RDA, the strong points are the fast application and simple generation of the major plot. Whereas the observed weak points are the relative difficult result interpretations and the time dependency over the entire analysis tasks. The latest can be neglected if the time series data contain the same number of frequency as recorded variables. On the other hand, the simpler process phase alignment and the result interpretation need to be highlighted as positive properties of the FBA. Whereas, the need for process understanding and the high time investment by deviation designation followed by subsequently feature extraction can be stated as weak points for this approach. Nevertheless, during the analysis, it was observed that the most challenging task was to identify meaningful variables and to draw the right conclusion out of the generated uni- and multivariate models. Furthermore, it was identified that it is an essential part to explain the hypotheses you are going to test for, as exact as possible before starting the data analysis.

### How to combine FBA and RDA

**Fig. 5:**
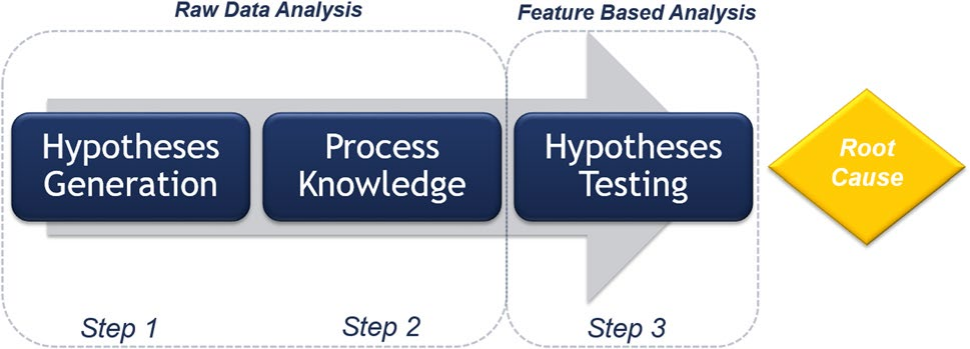
Root cause analysis (RCA) best practice workflow. This workflow is split into three steps, which will result in the RCA result using state-of-the-art data science tools. Each of these steps is mandatory to perform a RCA. Step 1 represents the need for generating reasonable hypotheses you are going to test for. Step 2 displays the implicit requirement of process knowledge, which can be gained by hypotheses generation, experience or process understanding. The required information to conduct these two steps can be collected out of the RDA comprehensively. Finally, the hypotheses need to be tested on significance and the optimal tool for this investigation is the FBA

To define appropriate hypotheses, the required process knowledge is often missing. On the other hand, if the process knowledge is already available, the major constraint might be the gap in statistical know-how to perform such an analysis. Within this investigation, we effectively found a solution to overcome these problems. While performing the RDA it was identified that it is very difficult to test on appropriate hypotheses. Additionally, it was observed that this approach will be an effective, fast and valuable tool to mine process information and to learn more about the entire process. As the FBA is focused on deviation within certain time frames, this tool will focus more on hypotheses testing than on generating process understanding. Moreover, within the FBA, process knowledge will help to extract reasonable deviations and interpret the generated results holistically. Concerning this investigation, we were able to provide a comprehensive roadmap, using well-known state-of-the-art statistical applications available in commercial software tools. To perform a RCA analysis either as process expert or as a statistic expert, we identified a hybrid solution by combining both approaches. With this new approach we will focus on applicability, to close the gap to perform an appropriated RCA and fulfill the requirements of regulatory agencies. Figure 5 displays the best practice workflow for performing a root cause analysis, by combing the investigated approaches.

### Suggested workflow

In Fig. 5 the suggested workflow on how to perform the optimal RCA using state-of-the-art data science tools is summarized. It can be seen that a combination of both approaches will result in the best practices of such an analysis. The presented workflow is split into three major parts, it was observed that each of them are essential to perform a reasonable and representative RCA and to address the variable which has the largest influence on a certain response variable. Step 1 describes the task of hypotheses generation. The aim of this step is to either brainstorm about the production process or to perform a RDA to identify the potentially most influencing variables on the response. Conducting this step will increase process knowledge and understanding. Steps 2 points to the major requirement of process knowledge. This awareness can be either collected by experience or by conducting the RDA as to not oversee any potential influence variables. Finally, Step 3 focuses on the testing of the predefined reasonable hypotheses and to detect significant variables influencing the response. The most appropriate tools for such testing are built in the FBA. This approach enables the extraction of certain time series information, regarding the hypotheses and to build multivariate model including these observations. The result can directly be transferred to a design of experiment approach which can be used to confirm the root cause and improve the biopharmaceutical production process.

### Application example

**Fig. 6:**
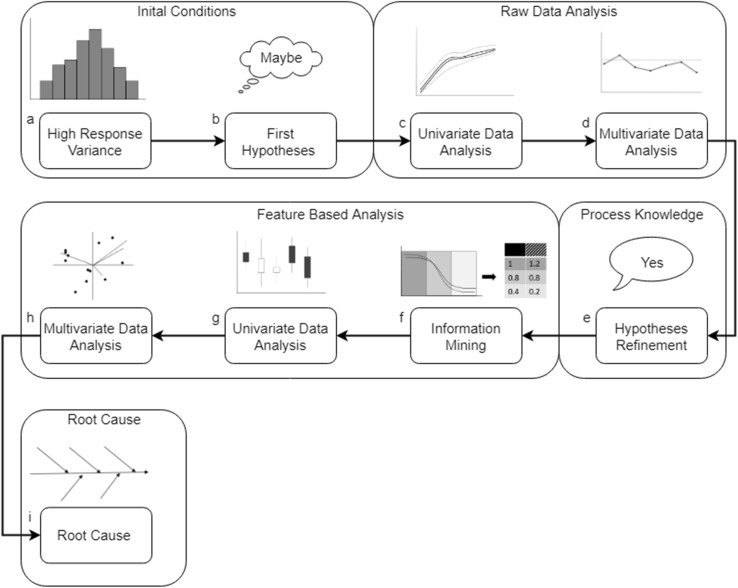
Schematic drawing of the suggested root cause analysis (RCA) workflow. **a** Initial observed variance of a response variable. **b** First assumption of causing this deviation due to process knowledge and/or literature review. The analysis workflow starts with the raw data analysis (RDA) especially with univariate analysis (**c**) followed by multivariate process evaluation, **d** the result of the RDA deepened the process understanding. This results in a better formulation of the final hypotheses for the reason of the observed differences (**e**). **f** First step of the feature bases analysis (FBA), the feature extraction due to the refined hypotheses. Univariate data analysis (**g**), and finally multivariate data analysis (**h**), result in a comprehensive conclusion about the reason for the response variance (**i**)

To evaluate and identify the root cause of a biopharmaceutical production process the suggested workflow can be applied as follows. A schematic drawing of the application of the workflow can be found n Fig. 6. Generally, the process is split into certain process phases and different time series data, online and offline, as well as discrete measurements have been collected during the process.

At the beginning of the analysis, a high variance of the response is observed (Fig. 6a) and the reason for this should be investigated. The data analyst already guesses the first hypotheses for this root cause due to process knowledge and literature review (Fig. 6b). To validate the first hypotheses and for a comprehensive process analysis, raw data analysis (RDA) is applied as the first step of the above-mentioned workflow.

Initially the collected data are investigated univariately (Fig. 6c). However, MVDA is required to observe abnormal variable behaviors and to create a gapless data matrix. Suspicious variables can be identified, are evaluated separately and removed from further analysis, if required. Subsequently performed MVDA (Fig. 6d) results in a better process understanding and identifies the significant variables causing the variance of the response. Additionally, this analysis step identifies the critical process phases responsible for the response difference.

With this result, the first hypotheses are refined and additional process knowledge leads to additional and valid hypotheses (Fig. 6e). At the next step, the Feature-Based Analysis (FBA) is performed. At this stage, we just know which variables cause the difference but not what event of a certain variable is responsible for this. The prior and new process understanding is used to extract this relevant information of the time series signal (Fig. 6f). The extracted data are investigated on data consistency and validity (Fig. 6g). The finally performed MVDA (Fig. 6h) depicts the significant variable events and their effect size causing the unknown response variance. This final result shows the coherent reason for the initial observed response deviation (Fig. 6i).

## Conclusion

The aim of this study was to compare the two major approaches currently used for performing a RCA and to reduce the gap between theoretical knowledge accumulation and practical utilization. We compared RDA, where the data are used holistically, with FBA, where deviations within the time series variables are investigated and information concerning the observed abnormalities are extracted.

- We observed that the required number of steps to conduct any of these approaches are almost identical whereas the accomplishment of these steps are different and the benefit is equal.
- We could successfully perform RCA by individually using both approaches and identified the strengths and weaknesses of each step within the approach.
- Finally, we suggested a new workflow applicable for every expert within a certain scientific discipline consisting of a combination of both approaches.
- This workflow will allow comprehensive science-based RCA which reduces the risk of performing an inappropriate RCA and fulfill the agency requirements.

## Abbreviations

CI: Confidence interval
CL: Control limits
FBA: Feature-based analysis
MVDA: Multivariate data analysis
PCA: Principal component analysis
PLS: Partial least square
RCA: Root cause analysis
RDA: Raw data analysis
RMSCV: Root mean square error of cross validation
STD: Standard deviation
USP: Upstream processing
